# Senolytic therapy reduces inflammation in epithelial cells from COPD patients and in smoke-exposure mice

**DOI:** 10.3389/fmed.2025.1451056

**Published:** 2025-04-28

**Authors:** Jonathan R. Baker, Leah Daly, Shyreen Hassibi, Genki Kimura, Yuki Nishimoto, Yasuo Kizawa, Kazuhiro Ito

**Affiliations:** ^1^National Heart and Lung Institute, Imperial College, London, United Kingdom; ^2^Department of Physiology and Anatomy, Nihon University School of Pharmacy, Funabashi, Japan

**Keywords:** COPD, senescence, senolytic, epithelial cells, SASP, inflammation, mice, air–liquid interface

## Abstract

**Introduction:**

Chronic obstructive pulmonary disease (COPD) is a disease of accelerated lung aging, with increased numbers of senescent cells found within the COPD Lung. Senescent cells may drive pathology by causing defective tissue repair and driving chronic inflammation via the release of inflammatory mediators known as the senescence-associated secretory phenotype (SASP). Senolytics are a new class of drugs that selectively remove senescent cells but have not previously been studied in COPD. We examined whether senescent cells are maintained during differentiation of COPD airway epithelial cells at the air–liquid interface and examined the role of the senolytic combination of dasatinib and quercetin on these cells and in a smoke-exposure mouse model.

**Methods:**

Non-smoker and COPD bronchial epithelial cells were differentiated at air–liquid interface (ALI). Senescence markers (p16^INKA^ and p21^WAF1^) were determined using Western blotting and SASP factors via Olink proteomics and Meso Scale Diagnostics (MSD). Cells and 11 days cigarette smoke (CS)-exposed mice were treated with the senolytic cocktail of dasatinib and quercetin (D + Q).

**Results:**

Increased senescence markers were maintained in COPD ALI epithelium when differentiated at air–liquid interface, and treatment with D + Q reduced senescence markers, proteases, and Th2 cytokines. Therapeutic oral treatment of D + Q to CS-exposed mice reduced senescence burden while reducing inflammatory cell infiltrates and mouse CXCL1.

**Conclusion:**

COPD subjects show increased airway epithelial senescence, and these cells can be cleared therapeutically using the senolytic cocktail of D + Q, reducing broad-spectrum pulmonary inflammation *in vitro* and *in vivo*.

## Introduction

Chronic obstructive pulmonary disease (COPD) is a chronic inflammatory lung disease that affects approximately 10% of people over the age of 45 years and is now ranked as the third most common cause of death (1). Inflammation occurs predominantly in the lung parenchyma and peripheral airways, resulting in irreversible and progressive airflow limitation due to parenchymal damage and airway narrowing (2). COPD is thought to be a disease of accelerated lung aging, with premature lung function decline (3, 4). One of the key hallmarks of aging is the accumulation of senescent cells (5), which are elevated in several cell types in the lungs of COPD subjects (6).

Cellular senescence is a state of stable cell cycle arrest that can be induced in response to various intrinsic and extrinsic stressors, causing a cell to lose its capacity to divide and aid in tissue repair (5). Senescent cells are not inert and remain metabolically active, secreting a combination of pro-inflammatory cytokines, chemokines, and matrix metalloproteinases, termed the “senescence-associated secretory phenotype” (SASP) (7). This pattern of inflammatory proteins closely mirrors the secretome of the COPD lung (8), suggesting that the SASP may be a key driver of chronic low-grade inflammation in COPD.

Many different cell types within the COPD lung are senescent, with elevated levels of senescent alveolar type II cells (9, 10), fibroblasts (11), small airway epithelial cells (12), and endothelial cells (10, 13). In mice, clearance of senescent cells *in vivo* via depletion of p16^INK4a^ both before and after exposure to cigarette smoke protects against smoke-induced emphysema, suggesting the importance of senescence in the pathology of the disease experimentally (9, 14). However, the pharmacological clearance of senescent primary COPD cells has not been well studied.

Currently, there are no disease-modifying therapies for the treatment of COPD; therefore, there is a great need to develop new treatments to slow disease progression. Senotherapies, consisting of senomorphics and senolytics, are novel therapeutics developed to specifically target senescent cells (15, 16). Senomorphics target the development of cellular senescence by inhibiting SASP release, while senolytics selectively kill senescent cells by reactivating apoptosis pathways and, therefore, do not affect healthy proliferating cells (16). One such senolytic treatment is the combination of dasatinib, a tyrosine kinase inhibitor, and quercetin, a flavonoid antioxidant (D + Q) (17). D + Q, as a senolytics combination, was identified by hypothesis-driven drug screens. Compounds were selected to inhibit the senescent cell anti-apoptotic pathways and induce apoptosis only in senescent cells (18). These compounds target BCL-2 family members, the PI3K pathway, and HIF-*α*, all of which are dysregulated in COPD and regulate resistance to apoptosis (19–21).

Monolayer cultures exposed to cigarette smoke extract and primary cells taken from COPD patients have routinely been utilized to understand the cellular and molecular mechanisms which drive COPD pathology while also being used as preclinical models (22). However, to better recapitulate the lung, especially the epithelial lining of the airways, epithelial cells grown at air–liquid interface can be utilized (23). These cultures have the advantage of being composed of ciliated cells, goblet cells, and basal cells, more recapitulating the cell types found within the respiratory tract. These cultures, therefore, have a greater resemblance to the lung and provide a better model to test novel therapeutic interventions.

COPD studies of senolytic treatment are lacking, but previous studies have shown that in *in vivo* and *ex vivo* bleomycin idiopathic pulmonary disease (IPF) models, D + Q selectively removed senescent cells, leading to reduced inflammation and increased lung function parameters in mice (24, 25). Pilot clinical studies of D + Q have shown that this senolytic cocktail is well tolerated and has no serious adverse effects, with further trials examining clinical outcomes needed (26, 27). We therefore examined the therapeutic clearance of senescent cells in bronchial epithelium differentiated at air–liquid interface and *in vivo* in a short-term smoke model using the senolytic combination of D + Q.

## Methods

### Primary human airway cells

MucilAir™ bronchial epithelium was provided fully differentiated at air–liquid interface as 24-well plate-sized inserts by Epithelix Sàrl. ALI cultures were maintained in 700 μL of MucilAir™ medium in the basal chamber and washed apically twice weekly to remove excess mucus until treatment (Supplementary Table 1 for demographics) (Geneva, Switzerland).

### BEAS2B D + Q experiments

BEAS2B cells were purchased from ATCC (Teddington, UK) and maintained in culture in keratinocyte media (Invitrogen, Paisley, UK) containing human recombinant epithelial growth factor (EGF) and bovine pituitary extracts (BPEs). Cells were stimulated for 72 h with 100 µM of H_2_O_2_ (Merck Life Science Limited, Dorset UK) before treatment with dasatinib (100 nM) and quercetin (50 µM) (Merk Life Science Limited, Dorset UK) for 24 h. Cells were then lysed for protein.

### Western blotting

Protein extracts were prepared by using RIPA buffer (150 mM NaCl, 1.0% IGEPAL CA-630, 0.5% sodium deoxycholate, 0.1% SDS, and 50 mM Tris, pH 8.0; MilliporeSigma) completed with protease inhibitor cocktail (Roche, Welwyn Garden City, United Kingdom). Protein extracts (40 μg) were analyzed by using SDS-PAGE (Thermo Fisher Scientific) and detected with Western blot analysis by chemiluminescence (ECL Plus; GE Healthcare, Hatfield, United Kingdom) on LiCor Fc imaging system. Band intensities were quantified by densitometry using the LiCor Image Studio Software. p16 Rabbit Antibody (ab108349) (Abcam), p21^Waf1/Cip1^ Mouse Antibody (DCS60) (#2946), Phospho-Histone H2A.X (Ser139) (20E3) Rabbit antibody (#9718), and Lamin B1 (D9V6H) Rabbit antibody (#13435) were used for human cells, while p21^Waf1/Cip1^ Rabbit (12D1) (#2947) (Cell Signalling Technologies) was used in mice. Anti-mouse IgG, HRP-linked Antibody (#7076), and Anti-rabbit IgG, HRP-linked Antibody (#7074) were used as secondary antibodies (Cell Signalling Technologies). Protein expression levels were expressed relative to *β*-actin (ab49900).

### Murine smoke model D + Q experiments

Specific pathogen-free A/J mice (male, 5 weeks old) were purchased from Sankyo Labo Service Co. Ltd. (Tokyo, Japan) and adapted for 1 week in a temperature (24 ± 1°C) and humidity (55 ± 5%) controlled room with a 12-h day-night cycle. The mice were reared on a standard diet and tap water *ad libitum*. Mice were exposed to cigarette smoke (4% cigarette smoke diluent with compressed air) for 30 min/d for 3 or 11 days using the commercially marketed filtered Hi-lite cigarettes (17 mg of tar and 1.4 mg of nicotine per cigarette; Japan Tobacco Inc) using a Tobacco Smoke Inhalation Experiment System for small animals (Model SIS-CS200; Sibata Scientific Technology Ltd) as described previously (23). Smoking was then ceased on day 11, and mice were treated with oral vehicle (10% PEG400 in saline) or oral dasatinib (5 mg/kg, PO) in combination with quercetin (50 mg/kg, PO) daily for 3 days. Mice were sacrificed, and bronchoalveolar lavage (BAL) fluid and lung tissue were collected. Each whole right lung was homogenized in 500 μL of Radioimmunoprecipitation assay (RIPA) buffer, completed with a protease inhibitor cocktail, and 40 μg of cell extracts were analyzed by sodium dodecyl sulfate-polyacrylamide gel electrophoresis (SDS-PAGE). Broncho-alveolar lavage fluid (BALF) was centrifuged at 500 × g for 10 min at 4°C. The cell pellet was resuspended in 0.2% sodium chloride (NaCl) to induce hemolysis of erythrocytes. After isotonization by adding the same volume of 1.6% NaCl, the total number of BAL cells was counted and aliquoted for flow cytometry, utilizing a Fluorescein isothiocyanate (FITC) conjugated anti-macrophage (MOMA2) antibody and anti-neutrophils (7/4) antibody (Acris Antibodies (GmbH, Herford, Germany) as previously described) (28). All animal studies were approved by the Ethics Review Committee for Animal Experimentation of Nihon University.

### qPCR

RNA was isolated from cells using the RNeasy mini kit (Qiagen Inc.) and reverse transcribed using the high-capacity cDNA reverse transcription kit (Life Technologies, UK). Quantification of mRNA expression [cdk2na (Mm00494449), cdkn1a (Mm04205640), tnf (Mm00443258), mmp12 (Mm00500554), and gnb2l1 (Mm01291968)] Taqman Assays were determined by real-time quantitative PCR (Applied Biosystems 7,500 Real-Time PCR System) using TaqMan gene expression master mix (Life Technologies, UK).

### Transepithelial electrical resistance

Transepithelial electrical resistance (TEER) was measured using a Millicell ERS-2 Voltohmmeter (Merck Life Science UK Limited). ALI cultures were washed for 10 min with PBS/media before reading TEER to remove any mucus. TEER was calculated by measuring the electrical impedance of the epithelium in OHMs; 139 was subtracted, as this was the baseline OHM of an insert without cells, and finally multiplied by 0.33, as this was the surface area of the trans well (0.33 cm^2^).

### Olink® proteomics

Olink® proteomics was performed on ALI culture apical wash. At time point 0, the apical side of the fully differentiated ALI cultures was washed in PBS for 10 min. Cells were then left for 72 h before being washed in 200 μL of media to collect inflammatory mediators released apically. Inflammatory proteins were measured using Olink® Proteomics Inflammation and Cardiovascular III Panel (184 inflammatory proteins) (Uppsala Sweden). Proteins are recognized by antibody pairs coupled to cDNA strands and extended by polymerase reaction.

### Meso scale diagnosis

CXCL8 and CCL5 were measured in the apical wash of ALI cultures using U-PLEX Human CXCL8 and CCL5 kits according to the manufacturer’s instructions (MesoScale Diagnostics, Maryland, USA).

### Proteome profiler array

Mouse bronchoalveolar fluid samples were collected and pooled from 6x room air, 6× 11 days smoke-exposed, and 6× 11 days smoke-exposed plus D + Q-exposed mice induction for a cytokine array analysis using the Proteome Profiler Mouse XL Cytokine Array (#ARY028. R&D Systems) according to manufacturer’s instructions. A signal was developed using an Odyssey Fc Imaging System. Band intensities were quantified by densitometry using the Li-Cor Image Studio Software.

### Statistical analysis

Data are expressed as means ± SEM. Results were analyzed using Mann–Whitney tests, Wilcoxon matched pairs signed-rank test, paired or non-paired Student’s *t*-tests, and Kruskal–Wallis with Bonferroni post-hoc test. GraphPad Prism 9 software (GraphPad Software, La Jolla, CA, USA) was used for analyses. Values of *p* < 0.05 were considered statistically significant.

## Results

### Increased senescent cells are maintained in ALI bronchial epithelium from COPD

**Figure 1:**
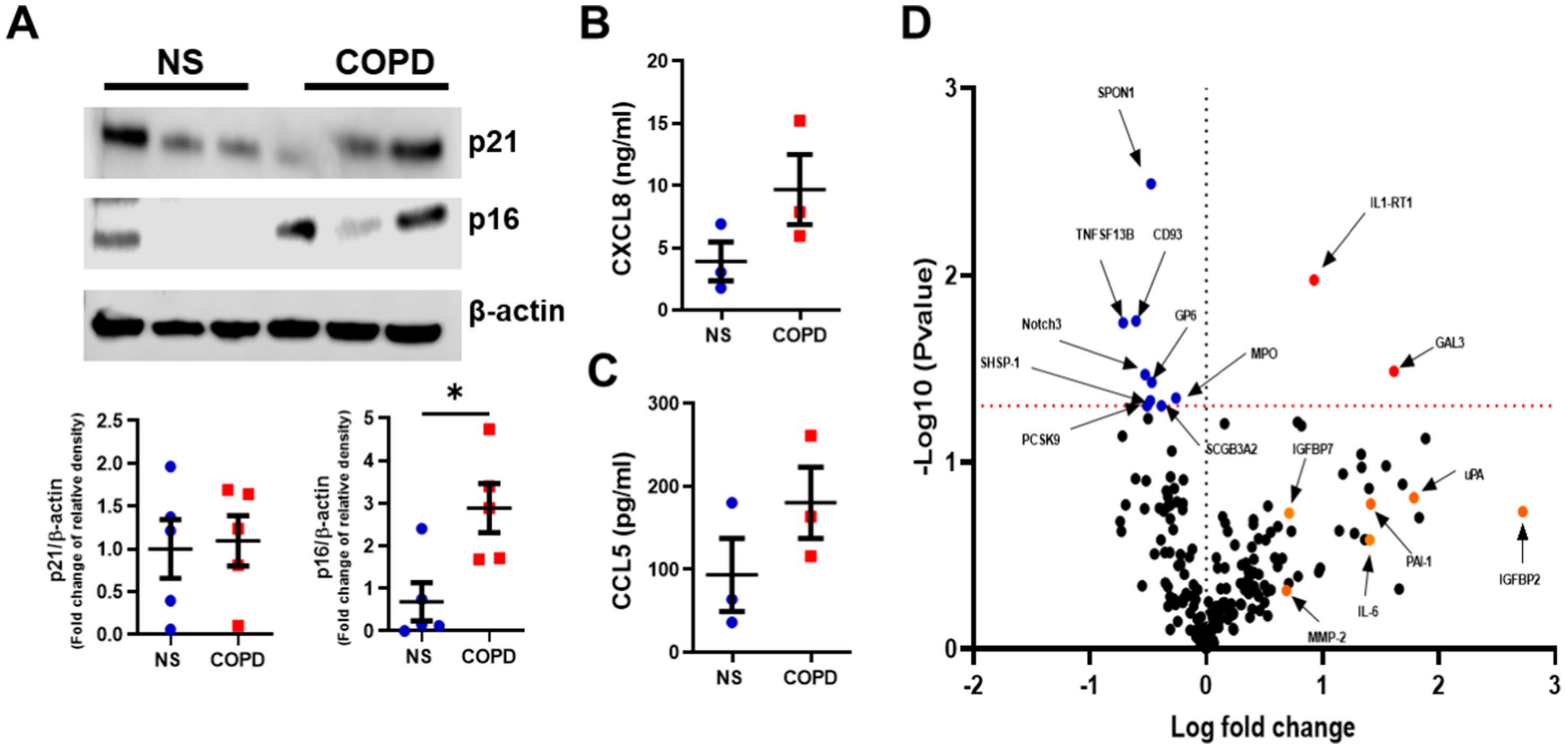
Elevated senescence markers are seen in well-differentiated airway epithelium of COPD subjects. **(A)** Representative Western blot image of p16^INK4a^ and p21^WAF1^ from NS (*n* = 5) and COPD (*n* = 5) Mucilair™ human airway epithelium cultured at air–liquid interface (image is of 3 NS and 3 COPD). **(B)** CXCL8 and **(C)** CCL5 release from basal apical wash from NS (*n* = 3) and COPD (*n* = 3) Mucilair™ human airway epithelium cultured at air–liquid interface, 72 h post apical wash. **(D)** Volcano plot of the Olink proteomics data comparing the baseline apical wash from NS (*n* = 3) and COPD (*n* = 3) Mucilair™ human airway epithelium cultured at air–liquid interface, 72 h post apical wash. Data are means ± SEM and were analyzed by using the Mann–Whitney U-test; *p < 0.05.

Increased levels of senescent cells have been reported in various cell types isolated from the COPD lung, with elevated levels of p16^INK4a^ and p21^WAF^, increased senescence associate-beta-galactosidase staining, SASP release, and elevated DNA damage markers being seen (12, 29–33). However, whether cellular senescence is detected and maintained in well-differentiated epithelium models is unknown. To assess this, we examined p16^INK4a^ and p21^WAF1^ protein levels in non-smoker and COPD epithelium differentiated at air–liquid interface (ALI). COPD subject ALI cultures had significantly more p16^INK4a^ protein expression than non-smokers, while p21^WAF1^ levels were not altered between groups (Figure 1A). Trends toward a decreased expression of Lamin B1 were also seen, but no evidence of increased levels of the DNA damage marker γH2AX was detected (Supplementary Figure 1). Next, we examined the inflammatory mediators released by these cells, examining the release of mediators apically from these cultures. CXCL-8 and CCL-5 trended to increased levels in the apical wash of cells from COPD subjects (Figures 1B,C). To fully understand the inflammatory profile of these cells and examine whether SASP factors were elevated, Olink® proteomics was performed on the apical wash of these cells. Of the 184 mediators examined, Galectin-3 (GAL3) and Interleukin 1 receptor, type I (IL-1RT1) (red in Figure 1D), were significantly elevated in the apical wash of the COPD subjects, while SPON1, TNSF13B, CD93, GP6, Notch3, SHSP-1, MPO, SCGB3A2, and PCSK9 (blue in Figure 1D) were significantly lower. Although not significant, many SASP factors trended toward an increase in IGFBP2, IGFBP7, PAI-1, uPA, IL-6, and MMP-2 in COPD ALI cultures (orange in Figure 1D), a full list of proteins examined and their log fold change and adjust *p*-values can be seen in Supplementary Table 1. These data suggest that a senescent phenotype is maintained in COPD airway epithelium during differentiation at ALI.

### Senolytic combination of D + Q reduces senescent cell burden and inflammatory mediators *in vitro*

**Figure 2:**
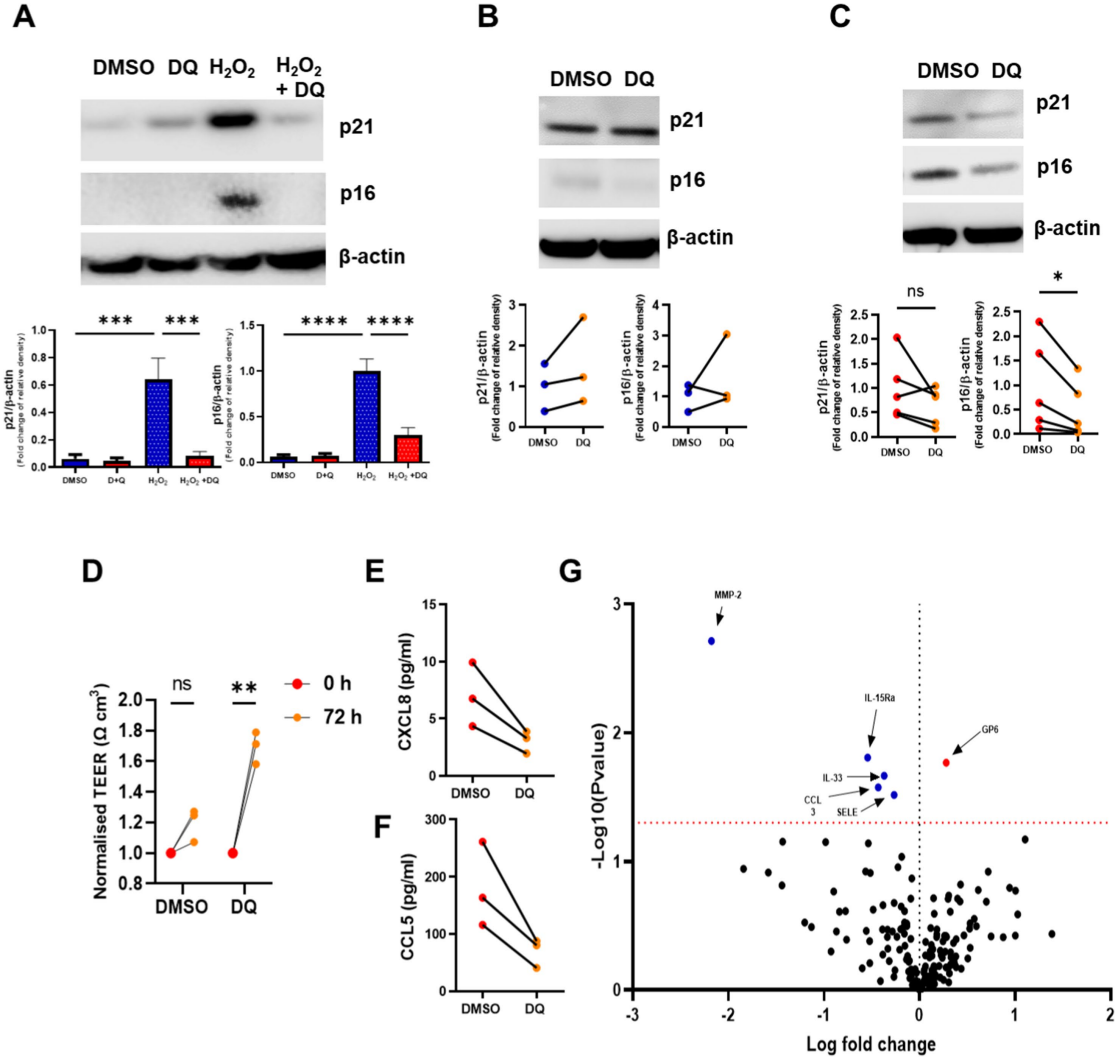
The senolytics combination of dasatinib and quercetin clears senescent airway epithelial cells and reduces inflammatory mediators. **(A)** Western blotting of p16^INK4a^ and p21^WAF1^ in BEAS2B cells stimulated H_2_O_2_ for 72 h and then treated with dasatinib and quercetin (D + Q) for 72 h. **(B)** Western blotting of p16^INK4a^ and p21^WAF1^ in NS (*n* = 3) Mucilair™ human airway epithelium cultured at air–liquid interface treated with and without dasatinib (100 nM) and quercetin (50 μM) for 72 h. **(C)** Western blotting of p16^INK4a^ and p21^WAF1^ in COPD (*n* = 5) Mucilair™ human airway epithelium cultured at air–liquid interface treated with and without D + Q. **(D)** Transepithelial resistance of COPD (*n* = 3) Mucilair™ human airway epithelium cultured at air–liquid interface measured prior and 72 h after DMSO or D + Q treatment. **(E)** CXCL8 and **(F)** CCL5 release from the apical wash of COPD (*n* = 3) Mucilair™ human airway epithelium cultured at air–liquid interface treated with and without D + Q. **(G)** Volcano plot of the Olink proteomics data comparing the apical was of COPD (*n* = 3) Mucilair™ human airway epithelium cultured at air–liquid interface treated with and without dasatinib and quercetin. Data are means ± SEM and were analyzed by using the Mann–Whitney U-test or unpaired Student’s t-test; *p < 0.05, **p < 0.01.

Next, we examined whether senescent cells could be cleared using the senolytic combination D + Q. We have previously shown that the H_2_O_2_ treatment of BEAS2B cells induces p16^INK4a^ and p21^WAF1^ (34), so we first examined whether D + Q could reduce these markers. BEAS2B cells were exposed to H_2_O_2_ for 72 h and then treated with D + Q for 72 h, leading to a significant reduction in H_2_O_2_-induced p16^INKa^ and p21^WAF1^ levels, suggesting the ability of this combination to clear senescent airway epithelial cells (Figure 2A). We next examined the effect of D + Q on ALI bronchial epithelium obtained from age-matched non-smokers, with these cells displaying lower expression of senescent marker p16^INK4a^, suggesting a lower senescent cell burden. D + Q had no effect on the levels of p16^INK4a^ or p21^WAF1^ (Figure 2B). However, when COPD ALI cultures were treated with D + Q, a significant (50%) (mean values of relative density of control relative to *β*-actin, DMSO 1.000 and D + Q 0.499) reduction in the protein level of p16^INK4a^ was observed (Figure 2C). This reduction in senescent cells was associated with increased cell integrity measured as the transepithelial resistance of the ALI cultures, suggesting an increase in the health of the epithelium (Figure 2D). The effect of D + Q was also assessed on inflammatory mediator release, with CXCL8 and CCL5 levels trending toward decrease (Figures 2E,F). Levels of other SASP factors were again assessed using Olink® proteomics and showed a significant increase in GP6 (red) being seen after treatment, while MMP-2, IL-15RA, IL-33, CCL3, and SELE were all significantly downregulated (Figure 2G); a full list of proteins examined and their log fold change and adjusted *p*-values can be seen in Supplementary Table 2.

### D + Q treatment clears senescence in a cigarette smoke mouse model

**Figure 3:**
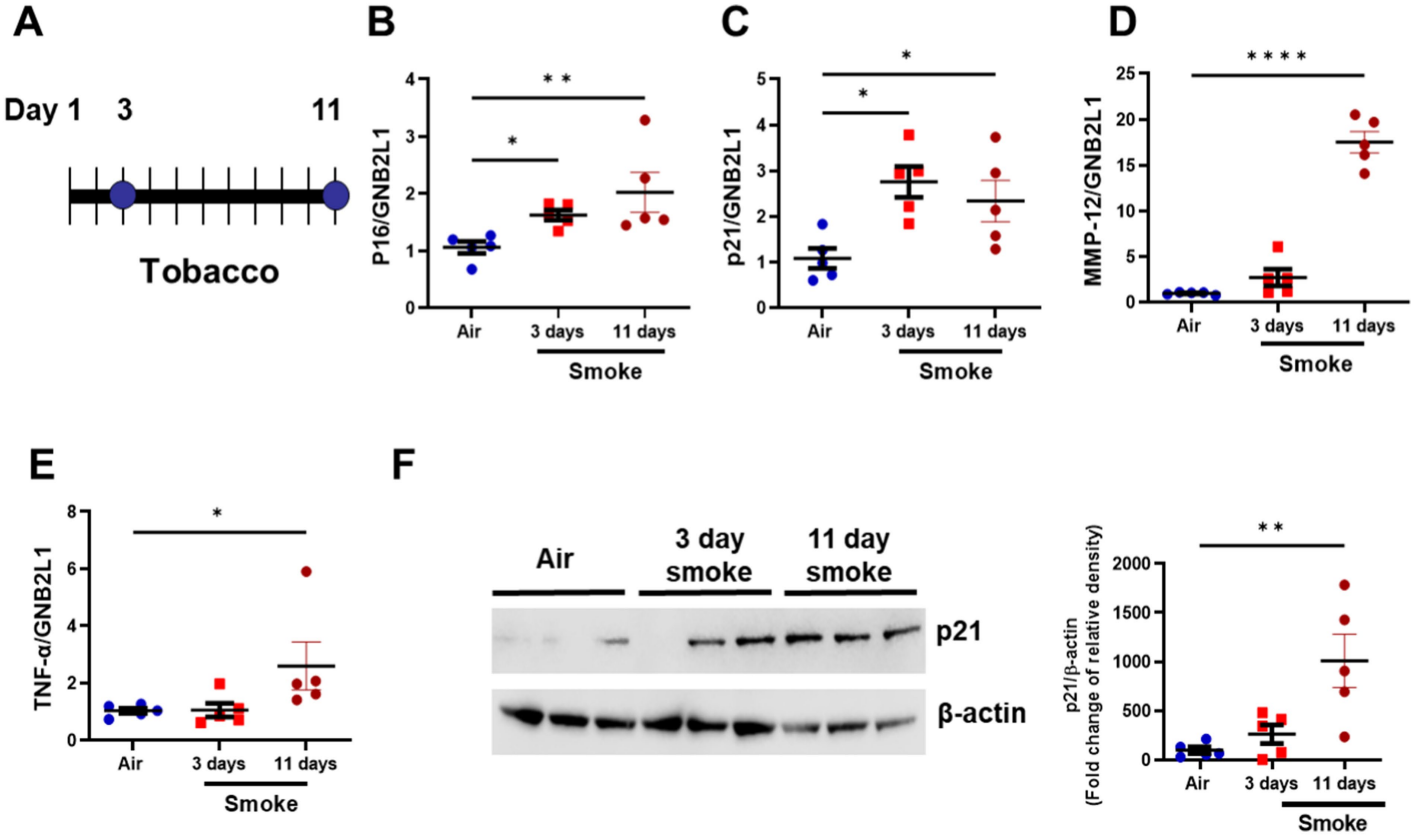
Short-term smoke exposure induces inflammation and senescence makers. **(A)** Diagram of 3- and 11-day tobacco smoke mouse. Gene expression of **(B)** p16^INK4a^ **(C)** p21^WAF1^ **(D)** MMP-12 and **(E)** TNF-*α* in lung homogenate samples from room air (11 day), 3- day and 11- day tobacco smoke exposed mice. **(F)** Representative Western blot of p21^WAF1^ in lung homogenate samples from room air (*N* = 5), 3- day (*N* = 5) and 11- day (*N* = 5) tobacco smoke-exposed mice (image is of lung homogenate from 3x air, 3× 3-day and 3× 11-day exposed mice). Data are means ± SEM and were analyzed by using the Kruskal–Wallis with *post hoc* Dunn’s multiple comparison tests; *p < 0.05, **p < 0.01, ****p < 0.0001.

To assess the effect of senolytic treatment *in vivo*, we utilized a cigarette smoke (CS) exposure mouse model. Both short- (35) and long-term (9) CS-exposed mice have elevated senescence markers, and therefore, we examined a short-term inflammatory smoke model we have used previously (36). Here, mice were exposed to smoke for 3 and 11 days (Figure 3A), with increased *Cdkn2a* and *Cdkn1a* gene expression levels detected at both time points, while increases in *Mmp-12* and *Tnf-α* were only seen at the 11-day time point (Figures 3B–E). The protein levels of p21^WAF1^ were only elevated in the 11 days smoke-exposed mice (Figure 3F). Due to the increase in p21^WAF1^ protein expression in the 11 days smoke mice, we used this model for D + Q treatment.

**Figure 4:**
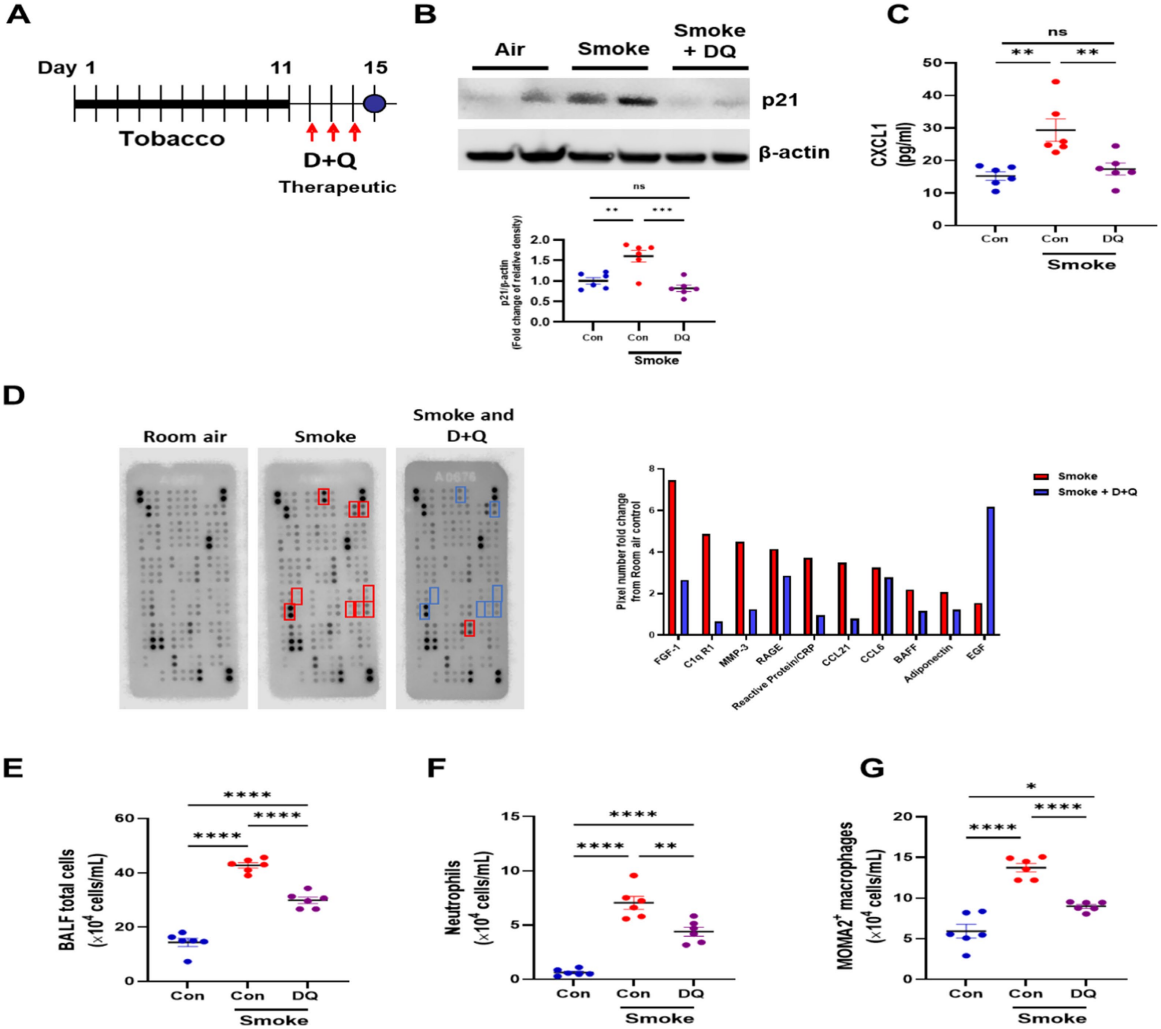
Dasatinib and quercetin treatments reduce senescence burden and inflammatory cell infiltrates in a smoke exposure mouse model. **(A)** Diagram of dasatinib and quercetin (D + Q) treatment in 11-day tobacco smoke mouse model. **(B)** Western blotting of p21^WAF1^ in lung homogenate samples from room air + oral vehicle 3 days (10% PEG400 in saline) (*N* = 6), 11-day tobacco smoke + oral vehicle 3 days (10% PEG400 in saline) (*N* = 6) and 11-day tobacco smoke with D + Q treated (*N* = 6) mice. **(C)** CXCL1 levels, **(D)** mouse proteome profiler array (fold change in pixels from room air-exposed mice), **(E)** total alveolar cells, **(F)** neutrophils, **(G)** macrophages in bronchoalveolar fluid from room air (*N* = 6), 11-day tobacco smoke + oral vehicle (10% PEG400 in saline) (*N* = 6), and 11-day tobacco smoke with D + Q treated (*N* = 6) mice. Data are means ± SEM and were analyzed by using the one-way ANOVA with post hoc Holm-Šídák’s multiple comparisons test or Kruskal–Wallis with post hoc Dunn’s multiple comparisons test; *p < 0.05, **p < 0.01, ****p < 0.0001.

Mice were exposed to CS for 11 days and then treated with D + Q for three consecutive days and then one final day of no treatment to allow for senescent cell clearance (Figure 4A); 11 days smoke significantly increased p21^WAF1^ protein levels as before, while treatment with D + Q significantly reduced p21^WAF1^ levels (Figure 4B). In the bronchiolar lavage fluid (BALF) of these mice, CXCL1 was significantly increased by CS exposure and reduced after D + Q treatment (Figure 4C). To gain further insight into the effects of D + Q treatment on inflammatory mediate release, a Proteome profiler array was performed on pooled BALF from room air, smoke, and smoke + D + Q exposed mice (Figure 4D). Fibroblast Growth Factor (FGF)-1, complement component C1q receptor (C1qR1), Matrix metalloproteinase (MMP)-3, receptor for advanced glycation end products (RAGE), C-reactive protein (CRP), chemokine ligand (CCL-)21, CCL6, B-cell activating factor (BAFF), and adiponectin were increased by at least 2-fold by cigarette smoke exposure compared to room air and all reduced by D + Q treatment. Interesting epidermal growth factor (EGF), which has been shown to suppress senescence, was induced after D + Q treatment (37); changes in all proteins can be seen in Supplementary Figure 2. Cigarette smoke significantly increased total alveolar lavage cell counts, with significant increases in both neutrophils and macrophages; these numbers are significantly reduced after D + Q treatment (Figures 4E,F).

## Discussion

COPD is associated with an accelerated aging phenotype, with increased senescent cells found in COPD lungs (4, 6). The accumulation of senescent cells may drive disease pathology by reducing the repair capacity of a cell via cell cycle arrest and drive chronic inflammation by releasing the SASP. Several clinical trials of senolytics are currently in progress, and senolytic treatment is seen to be anti-fibrotic and anti-inflammatory in IPF murine models (24, 25, 27). Here, we show that a senescent phenotype is maintained in COPD bronchial epithelial cells differentiated at air–liquid interface. Treatment of COPD ALI cultures with the senolytic cocktail of D + Q reduces senescence markers while also reducing inflammatory mediators and proteases. Therapeutic treatment of D + Q in cigarette smoke mouse model reduced neutrophil chemokine levels and inflammatory cell infiltrates into the lung. These data show that cellular senescence is elevated in the airway pseudostratified epithelium of COPD patients and a short-term CS exposure murine model. In addition, the clearance of these cells by a senolytic cocktail reduces inflammation in both *in vitro* and *in vivo* models.

Most *in vitro* work examining cellular senescence in COPD has been performed on primary cells grown at the monolayer. ALI cultures induce the differentiation of basal epithelial cells into a well-differentiated epithelium barrier consisting of basal, ciliated, and goblet cells (38). These models, therefore, provide a better translational model of the airway epithelium to test novel therapeutics, better recapitulating the *in vivo* epithelium (39). Our data suggest that senescence markers are maintained upon epithelium differentiation, suggesting that ALI cultures can be used as a model to study senescent cell clearance in COPD. These models have many advantages over monolayer culture. ALI cultures can be maintained in long-term culture, allowing the effects of drug/senolytic treatment to be examined over a longer period compared to submerged cultures, as well as having the ability to study phenotypical changes to the epithelium, such as mucus production and/or barrier integrity. The use of the ALI culture in testing novel senolytics has many advantages over monolayer cultures, but more work is needed to understand which epithelial cell types are senescent in this culture and what are the phenotypical changes in the epithelium after treatment. An additional advantage of the ALI model is the ability to sample from both the apical and basolateral sides of the cells, and as such, we measured the release of inflammatory mediators from the apical side of these cultures, mimicking what is released into the airway lumen. Although few SASP factors were significantly elevated in this experiment, large numbers of SASP factors did trend toward being increased, with this study not being powered to detect these changes. Furthermore, the ALI model can be treated from either the apical or basolateral sides of the cells, and we can mimic the treatment route of new medicines such as inhaled medicine and oral systemic drugs.

The removal of senescent cells has the potential to be a therapeutic treatment for many chronic diseases, including COPD. Clearance of p16^INK4a^ positive cells in an aging mouse model using a caspase-activated system led to a significant prolonging of lifespan (40). Genetic clearance of senescent cells before or after CS exposure *in vivo* reduced air space enlargement, suggesting clearance may both inhibit and reverse emphysema (9, 14). However, the therapeutic removal of these cells has not been studied in cells from the COPD lung. We utilized D + Q for these treatments as both are FDA-approved and therefore have increased translatability (14). Our data showed the ability to remove markers of senescence in the COPD airway epithelium differentiated at ALI. Interestingly, airway integrity also increased, suggesting an improvement in the epithelium layer after treatment. SASP factors, as well as senescence markers, were also significantly decreased. MMP-2 was the most significantly downregulated protein, which is elevated in COPD SAEC, suggesting the possibility that senolytic treatment may protect against airway remodeling and emphysema (12). IL-33 and CCL3 were also reduced, which could reduce Th2 inflammation and recruitment of inflammatory cells to the lung (41, 42). IL-15Ra was also significantly reduced, which is known to enhance IL-15 activity and increase NK and CD8 + T cell activation (43).

To assess the effects of D + Q *in vivo*, we utilized a short-term CS mouse model. In the optimization study, we found that CS exposure induces markers of senescence after 11 days of exposure but not after 3 days, while previously showing reduced levels of the putative anti-aging molecule sirtuin-1 (44). Previously, we showed that the induced pulmonary inflammation in the 3-day exposed mice resolved quickly, but in the 11-day model, the inflammation remained (28, 44). The 11-day CS exposure mice may therefore start to show signs of accelerated lung aging, but due to not being a chronic model, the mice did not show any signs of emphysema. Therapeutic treatment of the 11 days CS-exposed mice with D + Q reduced p21^WAF1^ protein and the neutrophil chemokine cxcl1, as well as further SASP facts indicated by the proteome array. These mice had reduced total cell counts in their BAL, with reduced numbers of neutrophils and macrophages, suggesting that clearance of senescent cells may reduce inflammatory cell recruitment to the lung. These data confirm previous findings showing that the clearance of senescent cells protects against phenotypes of the disease, but it is the first study to show this therapeutically (9). Future studies is needed to understand which cells become senescent within the lung after CS exposure and which are removed by D + Q treatment.

There are limitations to this study. First, the control and disease ALI-cultured epithelium were from a commercial source, and therefore, information on the patients’ demographics is limited. However, there is also an advantage to using these samples for preclinical drug screening. The ALI cultured epithelium is of high quality and reproducible (commercial standard), and the data are therefore reproducible. Second, Olink analysis was only performed on three NS and three COPD ALI apical washes. Changes in SASP factors in disease and after D + Q treatment may therefore have been missed due to low statistical power. However, this data gives insight into SASP factors that may be elevated senescent COPD epithelium and is being useful for future power calculations to determine the sample size for larger studies. Third, our data showed differences in senescence markers in the ALI cultures compared to monolayers. p21^WAF1^ levels were not elevated in COPD ALI cultures compared to controls but are increased in COPD airway epithelial cell monolayer cultures (12). This may be a caveat of the generation of ALI cultures in which p21^WAF1^ levels are upregulated during the differentiation; however, p21^WAF1^ is considered a marker of early senescence, and it is only upon upregulation of p16^INK4a^ that terminal senescence occurs. The COPD ALI cultures may, therefore, reflect a pool of terminal senescent cells compared to monolayer cultures (45). p21^WAF1^ is also elevated by contact inhibition in confluent monolayers, and as ALI cultures are a fully differentiated confluent layer of cells, this may explain these findings, suggesting p16^INK4a^ to be a better marker of senescence in ALI cultures. We also observed no increase in the DNA damage marker γH2AX in the COPD ALI cultures, and it may be that p21^WAF1^ is no longer being activated by the DNA damage response pathway and this also being a possible explanation for this not being upregulated (46). This is further validated by the fact that the first non-smoker sample in Figure 1A is from a 72-year-old, with this subject being the eldest individual of the non-smoking controls, and displays the highest non-disease p16^INK4a^ expression, potentially further validating p16 ^INK4a^ as a good senescence marker in these cultures. We do, however, show for the first time that senescent cells are elevated in COPD ALI differentiated epithelium models and can be cleared using the senolytic combination of D + Q, reducing inflammation. However, a more in-depth measure of senescence markers, such as Senescence Associated-*β*-Galactosidase staining, reactive oxygen species (ROS) levels, apoptosis markers, and further DNA damage markers, could be examined to fully understand the senescence phenotype in these ALI cultures and the differences of these to cells grown in monolayer.

Another limitation was our smoke model, which is a short-term smoke model with no emphysematous changes occurring, unlike long-term models. Previous *in vivo* COPD models using genetic ablation of senescent cells have used longer smoke exposure times (9, 14), with changes in lung structure as a readout. Further studies examining whether therapeutic treatment of D + Q in long-term-smoke models can lead to lung regeneration or slowing in lung function are needed to fully understand the consequence of senescent cell clearance in the treatment of COPD subjects. This model does, however, demonstrate that senescence can be induced after 11 days of smoke exposure and that this drives early pulmonary inflammation, which can be reduced with the removal of senescent cells. This model is, therefore, a potentially useful model to test the pharmacodynamics of novel senolytics. Finally, we could not detect protein levels of p16 in our mouse lung homogenate samples. This was due to not being able to find commercially available antibodies that could detect this protein. We, therefore, could not confirm, like the ALI model, a reduction of p16 at the protein level in our mouse model.

Overall, we show in two clinically translatable models that the clearance of senescent cells using senolytic therapeutics can reduce pulmonary inflammation and potentially reduce disease progression.

## Data Availability

The raw data supporting the conclusions of this article will be made available by the authors, without undue reservation.
