# Supplementary material for: Senolytic therapy reduces inflammation in epithelial cells from COPD patients and in smoke-exposure mice
Source: Front Med (Lausanne). 2025 Apr 28;12:1451056. doi: 10.3389/fmed.2025.1451056 (PMC12066254; doi:10.3389/fmed.2025.1451056)
Supplement: Supplementary file 1 [file Data_Sheet_1.docx]

**Supplementary materials**

|  | **Non-smoker**  **(N=5)** | **COPD**  **(N=5)** |
| --- | --- | --- |
| **Age (years)** | 57.2±22 | 68.1±8 |
| **Sex (M:F)** | 3/2 | 2/3 |

**Supplementary table 1. The characteristics of study subjects for ALI cultures**

COPD = chronic obstructive pulmonary disease. Data are expressed as mean value ± standard deviation.

| Mediator | Log fold change | Adjusted P.value |
| --- | --- | --- |
| SPON1 | -0.47462 | 0.003245 |
| IL-1RT1 | 0.925653 | 0.01058 |
| CD93 | -0.60368 | 0.017593 |
| TNFSF13B | -0.71484 | 0.018028 |
| Gal-3 | 1.614983 | 0.032683 |
| Notch 3 | -0.52488 | 0.034005 |
| GP6 | -0.4693 | 0.037418 |
| MPO | -0.26052 | 0.045415 |
| SHPS-1 | -0.48043 | 0.046932 |
| SCGB3A2 | -0.3858 | 0.050523 |
| PCSK9 | -0.50881 | 0.050787 |
| IL-18BP | -0.49971 | 0.058389 |
| CTSZ | 0.78421 | 0.061101 |
| IL-10RA | 0.157777 | 0.062301 |
| PI3 | 0.818617 | 0.06386 |
| vWF | -0.72115 | 0.072562 |
| Ep-CAM | 1.88641 | 0.074659 |
| DLK-1 | -0.2965 | 0.087341 |
| IL-6RA | 1.33303 | 0.090848 |
| ALCAM | 1.54682 | 0.104788 |
| TFF3 | 1.336423 | 0.107032 |
| CCL15 | 1.174647 | 0.115687 |
| PON3 | -0.30685 | 0.120023 |
| TNF-R2 | -0.60916 | 0.122689 |
| CPA1 | -0.19679 | 0.124536 |
| IGFBP-1 | -0.51757 | 0.12608 |
| CSTB | 1.687813 | 0.131767 |
| IL-22 RA1 | 1.40131 | 0.138497 |
| CCL3 | -0.27611 | 0.138552 |
| PECAM-1 | -0.34386 | 0.142763 |
| CD244 | -0.33038 | 0.153701 |
| uPA | 1.786293 | 0.155446 |
| OPN | -0.25173 | 0.163353 |
| FGF-21 | -0.20096 | 0.16662 |
| PAI | 1.416263 | 0.167394 |
| ADA | -0.6936 | 0.169352 |
| TNFB | -0.30702 | 0.169395 |
| TIMP4 | 0.5309 | 0.171775 |
| RETN | -0.33781 | 0.173557 |
| AXL | -0.40377 | 0.176474 |
| IL-1 alpha | -0.53043 | 0.177636 |
| NT-proBNP | -0.20348 | 0.179673 |
| CHIT1 | -0.36588 | 0.182623 |
| IGFBP-2 | 2.722283 | 0.184521 |
| IGFBP-7 | 0.71229 | 0.187725 |
| OSM | 0.14181 | 0.195857 |
| CXCL5 | 1.830197 | 0.198171 |
| ICAM-2 | -0.31023 | 0.201425 |
| CCL23 | 0.406493 | 0.203076 |
| LIF-R | -0.74003 | 0.207777 |
| IL33 | 0.164753 | 0.211658 |
| LDL receptor | 0.40574 | 0.215755 |
| TFPI | 0.61479 | 0.221582 |
| CDH5 | -0.2834 | 0.229862 |
| JAM-A | 1.14171 | 0.2331 |
| CSF-1 | -0.72793 | 0.234296 |
| MCP-1 | 0.731597 | 0.234474 |
| NRTN | 0.186167 | 0.234965 |
| MMP-10 | 0.554403 | 0.237181 |
| CXCL6 | 1.275343 | 0.240512 |
| IL4 | 0.4499 | 0.257481 |
| CASP-3 | 1.36534 | 0.26006 |
| LAP TGF-beta-1 | 0.511003 | 0.260358 |
| IL2-RA | -0.39083 | 0.260544 |
| IL6 | 1.406767 | 0.260657 |
| TR-AP | 0.246807 | 0.277106 |
| CST5 | 0.393663 | 0.284865 |
| CD8A | -0.12096 | 0.292588 |
| IL5 | -0.31498 | 0.304424 |
| TRANCE | -0.35706 | 0.304485 |
| MEPE | -0.44496 | 0.310094 |
| CCL4 | -0.22265 | 0.311965 |
| FGF-23 | -0.14603 | 0.321772 |
| SCF | 0.589837 | 0.32426 |
| CCL11 | 0.085427 | 0.325155 |
| FAS | 0.654527 | 0.327672 |
| DNER | 0.408983 | 0.330382 |
| EGFR | 0.342033 | 0.346568 |
| SELP | -0.22093 | 0.352453 |
| IL-10RB | 0.306447 | 0.354949 |
| OPG | -0.24266 | 0.35698 |
| CXCL16 | 0.61325 | 0.360966 |
| IL-12B | 0.095913 | 0.367224 |
| GDNF | 0.159977 | 0.367864 |
| MCP-3 | 0.980207 | 0.369046 |
| MCP-1 | 0.378917 | 0.380867 |
| TNFRSF10C | 0.363517 | 0.386368 |
| IL7 | 0.967507 | 0.386959 |
| IL-18R1 | 0.409457 | 0.390104 |
| NT-3 | -0.18804 | 0.398587 |
| uPA | 0.451847 | 0.398761 |
| CHI3L1 | 0.787943 | 0.409061 |
| CXCL9 | 0.36719 | 0.417996 |
| TNFRSF14 | 0.34236 | 0.43821 |
| SIRT2 | -0.35109 | 0.442666 |
| Gal-4 | 0.29477 | 0.447011 |
| t-PA | 0.708783 | 0.447249 |
| IL8 | -0.1117 | 0.455221 |
| TGF-alpha | 0.500327 | 0.458513 |
| CD40 | -0.55168 | 0.459157 |
| MCP-4 | 0.412463 | 0.463417 |
| IFN-gamma | -0.33051 | 0.465132 |
| IL-15RA | 0.173137 | 0.474968 |
| CCL16 | 0.124193 | 0.476441 |
| CCL20 | 1.65944 | 0.478073 |
| TNFSF14 | -0.07404 | 0.481664 |
| PLC | 0.33204 | 0.482989 |
| VEGFA | 0.5551 | 0.483894 |
| MMP-2 | 0.68952 | 0.487846 |
| MMP-9 | 0.52576 | 0.493228 |
| GRN | 0.43309 | 0.499952 |
| CCL24 | -0.17342 | 0.504978 |
| EPHB4 | 0.365467 | 0.50583 |
| PSP-D | -0.12381 | 0.521603 |
| MB | 0.449313 | 0.52295 |
| FABP4 | -0.31185 | 0.525933 |
| AXIN1 | -0.17932 | 0.525977 |
| ITGB2 | -0.27866 | 0.541021 |
| CD163 | -0.20231 | 0.542733 |
| IL-20 | -0.09181 | 0.544275 |
| FGF-5 | 0.01818 | 0.560892 |
| IL-1RT2 | -0.20079 | 0.56463 |
| RARRES2 | 0.501463 | 0.564916 |
| CPB1 | 0.158133 | 0.56581 |
| IL10 | 0.140043 | 0.570281 |
| CX3CL1 | 0.216237 | 0.574241 |
| STAMBP | 0.296687 | 0.576423 |
| TSLP | -0.26435 | 0.578059 |
| CTSD | 0.302417 | 0.579852 |
| CCL25 | -0.10894 | 0.589417 |
| FGF-19 | 0.087563 | 0.591006 |
| KLK6 | -0.32939 | 0.591644 |
| IL13 | 0.16761 | 0.595686 |
| AZU1 | 0.157293 | 0.60384 |
| IL18 | 0.395623 | 0.613941 |
| GDF-15 | 0.46101 | 0.622327 |
| TR | 0.09441 | 0.639141 |
| CD5 | 0.038827 | 0.639414 |
| IL-24 | -0.13817 | 0.641627 |
| MCP-2 | 0.235343 | 0.645238 |
| Flt3L | 0.19626 | 0.646459 |
| TNF-R1 | 0.228943 | 0.65452 |
| MMP-1 | 0.52793 | 0.665365 |
| PDGF subunit A | 0.083203 | 0.668056 |
| SELE | -0.0477 | 0.674 |
| CCL19 | 0.116123 | 0.675961 |
| IL-20RA | 0.375303 | 0.677264 |
| PGLYRP1 | 0.051433 | 0.681213 |
| IL-2RB | 0.120363 | 0.688168 |
| CNTN1 | -0.07938 | 0.691262 |
| CCL28 | 0.187343 | 0.69296 |
| BLM hydrolase | 0.104903 | 0.705149 |
| CXCL11 | -0.24524 | 0.71192 |
| IL-17RA | 0.104253 | 0.716519 |
| SLAMF1 | 0.059053 | 0.73428 |
| TRAIL | 0.10781 | 0.766995 |
| EN-RAGE | -0.02331 | 0.769321 |
| AP-N | -0.07252 | 0.777765 |
| OPG | 0.23309 | 0.778999 |
| PRTN3 | 0.032813 | 0.782869 |
| PD-L1 | -0.30883 | 0.786806 |
| LIF | 0.236277 | 0.789335 |
| LTBR | 0.053113 | 0.793358 |
| U-PAR | -0.13838 | 0.793467 |
| TLT-2 | -0.08367 | 0.807156 |
| CXCL1 | -0.04279 | 0.825467 |
| CASP-8 | -0.07653 | 0.836107 |
| CDCP1 | -0.09026 | 0.851778 |
| COL1A1 | -0.02405 | 0.857404 |
| TNF | -0.08596 | 0.865533 |
| Beta-NGF | 0.01005 | 0.881262 |
| CD6 | 0.031307 | 0.885735 |
| 4E-BP1 | 0.05932 | 0.900249 |
| IL-17A | -0.05166 | 0.906543 |
| MMP-3 | -0.05452 | 0.925975 |
| IL-17C | 0.064997 | 0.9325 |
| TNFRSF9 | 0.009777 | 0.945439 |
| TWEAK | 0.02542 | 0.945985 |
| ARTN | 0.045293 | 0.950626 |
| CXCL10 | 0.037357 | 0.957918 |
| ST2 | -0.03599 | 0.958327 |
| HGF | 0.008433 | 0.984721 |
| IL2 | 0.003123 | 0.984866 |
| ST1A1 | -0.00738 | 0.990953 |

**Supplementary table 2. Log fold change data for OLINK analysis of Non-smoker v COPD apical media from ALI cultures.** Log fold change and p value for all 184 mediators analysed in the ALI culture apical wash.

| Mediator | Log fold change | Adjusted P.value |
| --- | --- | --- |
| MMP-2 | -2.17794 | 0.001935 |
| IL-15RA | -0.54354 | 0.015531 |
| GP6 | 0.278083 | 0.017068 |
| IL33 | -0.37113 | 0.021569 |
| CCL3 | -0.43331 | 0.026552 |
| SELE | -0.26639 | 0.030347 |
| TWEAK | 1.104123 | 0.06753 |
| CXCL9 | -1.43303 | 0.070153 |
| IL4 | -0.98429 | 0.070704 |
| CCL4 | -0.53649 | 0.072142 |
| FGF-5 | -0.1882 | 0.092146 |
| CCL11 | -0.22683 | 0.110802 |
| CXCL10 | -1.8439 | 0.114138 |
| PI3 | -0.56566 | 0.11964 |
| ADA | 0.71742 | 0.119977 |
| CXCL11 | -1.58302 | 0.121896 |
| MMP-10 | -0.51657 | 0.122867 |
| ITGB2 | -0.08134 | 0.1356 |
| IL-18R1 | 0.428397 | 0.151298 |
| LIF | -1.441 | 0.153735 |
| 4E-BP1 | 0.94251 | 0.160163 |
| STAMBP | 0.612797 | 0.167122 |
| CDCP1 | 1.003393 | 0.168635 |
| MCP-1 | -0.89877 | 0.171381 |
| CHIT1 | 0.304737 | 0.184553 |
| MPO | 0.145837 | 0.193194 |
| PGLYRP1 | 0.396413 | 0.194518 |
| Beta-NGF | -0.0931 | 0.194562 |
| TSLP | 0.298617 | 0.195127 |
| RETN | 0.42291 | 0.204674 |
| TFF3 | 0.697897 | 0.206024 |
| OPN | -0.26708 | 0.20997 |
| FGF-19 | -0.38425 | 0.218606 |
| NRTN | -0.19314 | 0.22491 |
| GDNF | -0.48536 | 0.237569 |
| MCP-1 | -0.77597 | 0.243357 |
| SPON1 | -0.15818 | 0.243488 |
| SIRT2 | 0.320793 | 0.245803 |
| U-PAR | -0.83805 | 0.246394 |
| TNFSF14 | 0.15238 | 0.255209 |
| PRTN3 | 1.026783 | 0.258393 |
| DNER | 0.571673 | 0.279977 |
| uPA | -1.20183 | 0.298163 |
| CCL19 | -0.15693 | 0.299021 |
| ST1A1 | 0.527223 | 0.302732 |
| CCL16 | -0.13522 | 0.303875 |
| CD6 | -0.13297 | 0.317303 |
| IL18 | 0.593697 | 0.318479 |
| MCP-3 | -1.13133 | 0.32329 |
| AXL | -0.17641 | 0.329212 |
| Notch 3 | 0.115997 | 0.329799 |
| TR-AP | 0.522913 | 0.333443 |
| CCL23 | -0.1978 | 0.336837 |
| SCGB3A2 | 0.180763 | 0.338085 |
| Gal-4 | -0.38957 | 0.338598 |
| MB | -0.33656 | 0.339014 |
| TNFRSF9 | -0.55899 | 0.347481 |
| IL-17C | -0.87115 | 0.350618 |
| IGFBP-2 | -0.28433 | 0.350922 |
| IL-10RA | -0.1601 | 0.365228 |
| IL6 | 1.385577 | 0.366006 |
| TNFSF13B | 0.179767 | 0.369546 |
| LDL receptor | 0.46303 | 0.375829 |
| AZU1 | 1.001893 | 0.377244 |
| CST5 | 0.261343 | 0.379072 |
| IL-24 | 0.277167 | 0.380097 |
| PLC | 0.74787 | 0.383525 |
| IL10 | -0.23691 | 0.385619 |
| PSP-D | 0.17823 | 0.387108 |
| CTSZ | 0.281553 | 0.387192 |
| CX3CL1 | -0.33815 | 0.387199 |
| CD40 | 0.875 | 0.388293 |
| IL-12B | -0.09198 | 0.400476 |
| EPHB4 | 0.38325 | 0.402542 |
| PAI | -0.76408 | 0.406204 |
| uPA | -0.52286 | 0.416647 |
| IL-17A | 0.244333 | 0.417482 |
| SHPS-1 | 0.06283 | 0.422378 |
| LIF-R | 0.354803 | 0.430728 |
| NT-proBNP | 0.12384 | 0.446124 |
| IL-1 alpha | -0.15435 | 0.453176 |
| Gal-3 | 0.52835 | 0.469911 |
| ST2 | -0.33291 | 0.476481 |
| IL-18BP | 0.13812 | 0.481264 |
| IL2-RA | -0.20913 | 0.486409 |
| MEPE | 0.216987 | 0.493976 |
| PCSK9 | 0.110677 | 0.499472 |
| vWF | 0.1099 | 0.501509 |
| CXCL5 | -0.92762 | 0.502794 |
| FABP4 | 0.26779 | 0.50359 |
| TIMP4 | 0.324607 | 0.509859 |
| AXIN1 | 0.235133 | 0.5164 |
| KLK6 | -0.38522 | 0.529566 |
| CASP-8 | 0.22476 | 0.53618 |
| CCL25 | 0.061397 | 0.538714 |
| CD8A | 0.129947 | 0.542972 |
| IL-17RA | -0.12643 | 0.543088 |
| EGFR | 0.378567 | 0.548546 |
| Flt3L | 0.328497 | 0.555952 |
| TR | 0.1043 | 0.55905 |
| IGFBP-1 | -0.12665 | 0.559409 |
| TNFRSF10C | 0.238383 | 0.561474 |
| MMP-3 | 0.361667 | 0.566556 |
| EN-RAGE | 0.50504 | 0.568207 |
| IL8 | -0.13803 | 0.578101 |
| IL-20 | -0.10672 | 0.591913 |
| TNF-R1 | -0.3188 | 0.595727 |
| FGF-23 | -0.11384 | 0.601865 |
| CXCL16 | 0.360577 | 0.606176 |
| PD-L1 | -0.52072 | 0.618076 |
| IL7 | 0.32649 | 0.632451 |
| CSTB | 0.37254 | 0.643385 |
| CSF-1 | 0.204903 | 0.650241 |
| IL5 | 0.149963 | 0.652575 |
| IL-1RT1 | 0.310987 | 0.65413 |
| CD93 | 0.061863 | 0.656608 |
| LTBR | 0.16352 | 0.663297 |
| CHI3L1 | 0.381803 | 0.664667 |
| HGF | 0.276853 | 0.666961 |
| MMP-1 | -0.60063 | 0.676847 |
| OPG | 0.43525 | 0.682417 |
| PDGF subunit A | -0.04645 | 0.688668 |
| IL2 | -0.08901 | 0.693019 |
| TGF-alpha | -0.25844 | 0.701458 |
| CD5 | 0.04645 | 0.701874 |
| TNFB | 0.128487 | 0.702588 |
| IL13 | -0.11452 | 0.717764 |
| CCL15 | -0.09272 | 0.720404 |
| CNTN1 | 0.04538 | 0.734224 |
| PECAM-1 | -0.03142 | 0.735071 |
| FAS | 0.311403 | 0.742395 |
| TNFRSF14 | 0.162653 | 0.755859 |
| CXCL6 | -0.082 | 0.75998 |
| IL-6RA | 0.145923 | 0.765841 |
| TFPI | 0.124543 | 0.767286 |
| CCL24 | 0.088353 | 0.787754 |
| MMP-9 | -0.26852 | 0.790847 |
| MCP-4 | 0.215273 | 0.799496 |
| IL-20RA | -0.10287 | 0.809186 |
| IGFBP-7 | 0.155 | 0.811996 |
| OPG | 0.055843 | 0.812414 |
| CTSD | 0.129483 | 0.812635 |
| CPB1 | 0.09632 | 0.819734 |
| t-PA | 0.25082 | 0.834871 |
| NT-3 | -0.03793 | 0.850879 |
| PON3 | -0.03054 | 0.853117 |
| CCL20 | -0.41061 | 0.85315 |
| LAP TGF-beta-1 | -0.04619 | 0.85452 |
| ARTN | 0.10292 | 0.854879 |
| SLAMF1 | 0.043787 | 0.859888 |
| DLK-1 | 0.035923 | 0.872127 |
| Ep-CAM | 0.304907 | 0.873445 |
| TNF-R2 | 0.05201 | 0.875012 |
| SELP | 0.03527 | 0.877845 |
| IL-1RT2 | -0.05227 | 0.881162 |
| CXCL1 | -0.02393 | 0.887177 |
| GDF-15 | 0.11783 | 0.895987 |
| CASP-3 | 0.15674 | 0.89619 |
| COL1A1 | -0.01755 | 0.89876 |
| RARRES2 | 0.091657 | 0.901936 |
| GRN | 0.08497 | 0.902202 |
| CD163 | -0.03136 | 0.90902 |
| TNF | -0.07597 | 0.913339 |
| AP-N | 0.028793 | 0.915923 |
| MCP-2 | 0.104733 | 0.920987 |
| BLM hydrolase | 0.029393 | 0.923897 |
| JAM-A | 0.128457 | 0.934395 |
| TLT-2 | -0.02724 | 0.935897 |
| VEGFA | -0.05382 | 0.952168 |
| TRAIL | 0.018127 | 0.953161 |
| FGF-21 | 0.008363 | 0.953647 |
| ICAM-2 | -0.00793 | 0.956483 |
| SCF | -0.03935 | 0.957999 |
| CCL28 | -0.02681 | 0.958616 |
| IL-22 RA1 | -0.01707 | 0.960742 |
| CPA1 | 0.00701 | 0.962036 |
| CDH5 | -0.00684 | 0.969915 |
| IL-10RB | -0.0118 | 0.970853 |
| IL-2RB | 0.014777 | 0.974298 |
| CD244 | -0.00595 | 0.97473 |
| TRANCE | 0.009747 | 0.976304 |
| ALCAM | 0.024467 | 0.978253 |
| OSM | 0.001427 | 0.985495 |
| IFN-gamma | -0.00822 | 0.988773 |

**Supplementary table 3. Fold change data for OLINK analysis of COPD DMSO and D+Q apical media from ALI cultures.** Log fold change and p value for all 184 mediators analysed in the ALI culture apical wash.


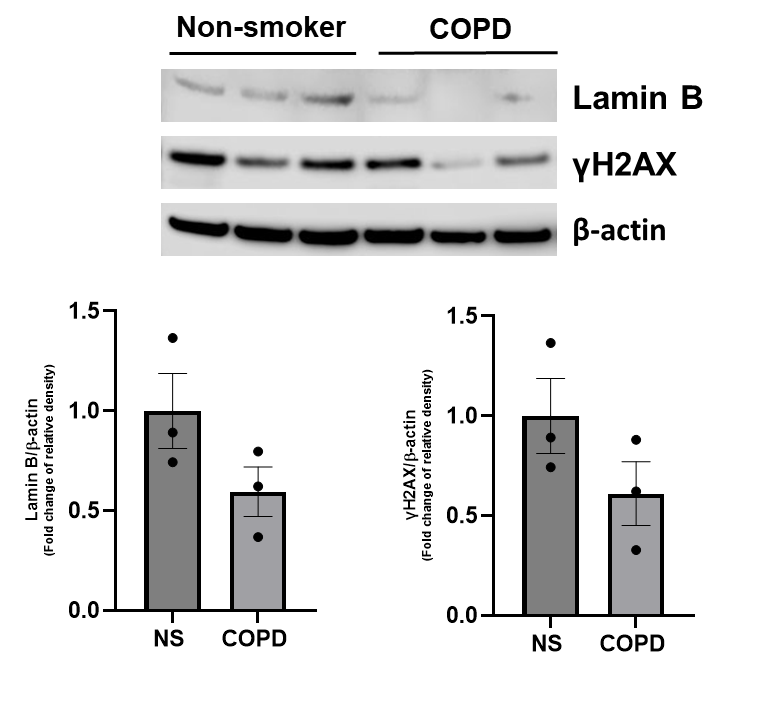


Supplementary Figure 1. **Examination of further senescence markers in COPD ALI cultures.** Representative Western blotting image of Lamin B and γH2AX from NS (n=3) and COPD (n=3) MucilairTM human airway epithelium cultured at air liquid interface.

Supplementary Figure 2. Heatmap of Proteome Profiler Mouse XL Cytokine Array data. Data are presented as fold change from room air exposed mice.
